# Another Death from Chloroform

**Published:** 1863-09

**Authors:** 


Another Death from Chloroform.—We wonder, as nearly every English journal brings to us the account of still another death from chloroform, how many sacrifices will yet be required to open the eyes of the public there, for of the profession we have no hope, to the facts that chloroform may kill anybody who inhales it, while ether is capable of producing the same anæsthetic effect with only a little more trouble, but with entire safety. The last victim was a distinguished member of our own profession, Mr. F. Wakefield Skey, M.R.C.S., the following account of whose death is taken from the *Lancet* of June 6th.

"The profession to whom he is so deservedly well known will sympathize with Mr. Skey in the severe loss sustained by him in the death of his eldest son, which took place on Friday last under the following melancholy circumstances: The deceased had long suffered from a severe neuralgic disease, to obtain relief from which he frequently had recourse to chloroform, but with only temporary benefit. More than once he carried the anæsthesia to such an extent as to cause considerable alarm, especially on one occasion, when, acting as house-surgeon to St. Bartholomew's Hospital, he remained so long under the influence of the subtle agent, as to necessitate recourse to galvanism and to artificial respiration. On recovery he assured his anxious and deeply afflicted parents that it was the last time he would use it without advice. The promise he religiously kept until Friday last, when an unusually severe paroxysm came on, and having to meet his father and several friends at dinner, he again had recourse to his old, but on this occasion fatal, remedy. How long he had succumbed to the active agent is not precisely known, but he was found in his chamber, kneeling at a chair, with a handkerchief to his face, which had contained the chloroform. He immediately received every attention, but too late; the vital spark could not be recalled. The deceased, who was only 31, had attained to considerable excellence in music, poetry and painting."

